# Restoration of a large osteochondral defect of the knee using a composite of umbilical cord blood-derived mesenchymal stem cells and hyaluronic acid hydrogel: a case report with a 5-year follow-up

**DOI:** 10.1186/s12891-017-1422-7

**Published:** 2017-02-02

**Authors:** Yong-Beom Park, Chul-Won Ha, Choong-Hee Lee, Yong-Geun Park

**Affiliations:** 1 Department of Orthopedic Surgery, Chung-Ang University Hospital, Chung-Ang University College of Medicine, 102 Heukseok-ro, Dongjak-gu, Seoul 06973 South Korea; 2 Department of Orthopaedic Surgery, Samsung Medical Center, Sungkyunkwan University School of Medicine, 81 Irwon-ro, Gangnam-gu, Seoul, 06351 South Korea; 3 Stem Cell & Regenerative Medicine Institute, Samsung Medical Center, 81 Irwon-ro, Gangnam-gu, Seoul, 06351 South Korea; 4 Department of Health Sciences and Technology, SAIHST, Sungkyunkwan University, Seoul, South Korea; 5 Department of Orthopedic Surgery, Jeju National University Hospital, Jeju National University School of Medicine, 15 Aran 13-gil, Jeju-si, 63241 South Korea

**Keywords:** Knee, Large osteochondral defect, Umbilical cord blood, Mesenchymal stem cell, Hyaluronic acid

## Abstract

**Background:**

The treatment of articular cartilage defects is a therapeutic challenge for orthopaedic surgeons. Furthermore, large osteochondral defects needs restoration of the underlying bone for sufficient biomechanical characteristics as well as the overlying cartilage.

**Case presentation:**

A symptomatic large osteochondral defect in the knee joint was restored using a composite of umbilical cord blood-derived mesenchymal stem cells (UCB-MSCs) 0.5 x 10^7^/ml and 4% hyaluronic acid (HA) hydrogel. Significant improvements in pain and function of the knee joint were identified by the evaluation at 12 months after surgery. A hyaline-like cartilage completely filled the defect and was congruent with the surrounding normal cartilage as revealed by magnetic resonance imaging (MRI), a second-look arthroscopy and histological assessment. The improved clinical outcomes maintained until 5.5 years. MRI also showed the maintenance of the restored bony and cartilaginous tissues.

**Conclusion:**

This case report suggests that the composite of allogeneic UCB-MSCs and HA hydrogel can be considered a safe and effective treatment option for large osteochondral defects of the knee.

## Background

The treatment of articular cartilage defects continues to be one of the most challenging clinical problems for orthopaedic surgeons. When isolated chondral or osteochondral defects are left untreated, they do not heal and may progress to symptomatic degeneration of the joint [1]. Therefore, early surgical intervention for symptomatic lesions which are not responding to conservative treatment is often suggested in an effort to restore normal joint congruity and pressure distribution, and to prevent further injury. Therefore, several techniques for cartilage restoration have been developed [2–4]. Microfracture, osteochondral autograft transfer (OAT) and autologous chondrocyte implantation (ACI) are the commonly applied methods, which will be introduced more in detail below regarding the case of this paper.

The treatment of large osteochondral defects involving the cartilage as well as the subchondral bone is more challenging because of two different tissues with different healing potential [5]. Microfracture, a bone marrow stimulating arthroscopic technique, seems to be the most frequently used method to repair small sized articular cartilage defects (<2 cm^2^) [6], however, it is generally not recommended for osteochondral defects due to limited potential for restoring the underlying bony tissue [7]. OAT offers the advantage of restoring cartilage tissue as well as subchondral bony tissue. However, limited graft availability and donor site morbidity are major limitations [8]. Furthermore, uneven surface or unstable fixation in multiple grafting for a large defect is also a concern [9]. Large osteochondral defects can sometimes be treated by ACI, however ACI is a two-staged procedure and it is hard to apply the graft in lesions with deep (more than 6 to 8 mm) subchondral defects [10]. ACI is also known to have very limited potential in restoring bony tissues and often requires bone grafts for subchondral bone restoration in cases of large osteochondral defects [11, 12]. Osteochondral allograft is an another possible option, but the limited availability of fresh allograft is a major drawback in clinical practice [13]. Therefore, there still lacks an optimal method to restore the cartilaginous and bony tissue in a large osteochondral defect.

Recently, mesenchymal stem cells (MSCs) have become attractive as one of the potential candidates for cellular therapy, featuring self-renewal, proliferation and differentiation into mesenchymal tissues, including bone, tendon, muscle and cartilage [14]. Moreover, MSCs likely exhibit a capacity of immune-tolerance or immune modulation that may allow allogeneic MSCs transplantation feasible [15]. There are only two reports in the literature on the effect of autologous MSCs for osteochondral defect of the knee [16, 17]. We, however, could not find a report of allogeneic MSCs transplantation for the restoration of osteochondral defect. In addition, it was hardly investigated whether MSCs were effective to treat large osteochondral defect. Umbilical cord blood-derived MSCs (UCB-MSCs) are ease to obtain, are non-invasively collected, and have a good expansible capacity [18, 19]. In addition, some studies suggest immunomodulatory effects [20, 21]. Therefore, UCB-MSCs can be an appropriate source for allogeneic transplantation.

We previously reported that transplanting of UCB-MSCs and hyaluronic acid hydrogel composite resulted in favorable cartilage repair in animal models [22–26]. Moreover, recently, we demonstrated that transplantation a composite of allogeneic UCB-MSCs and HA hydrogel was safe and effective modality for cartilage repair in osteoarthritic knees, which was followed up for more than 7 years without any significant adverse events [27]. In this paper, we report a first case of transplanting a composite of allogeneic UCB-MSCs and HA hydrogel in large osteochondral defect.

## Case presentation

**Fig. 1:**
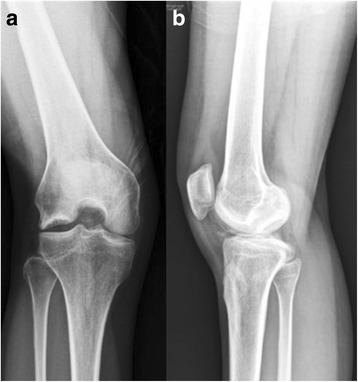
**a, b** Simple radiographic images of a 31-year-old female showed a large osteochondral defect on the lateral femoral condyle of right knee

**Fig. 2:**
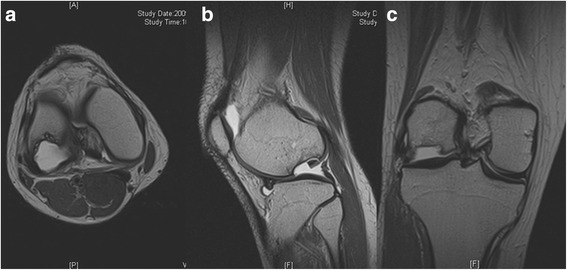
Preoperative magnetic resonance image. **a** Axial, **b** sagittal and **c** coronal images showed large osteochondral defect (approximately 2.7 cm × 2.2 cm sized and 1.5 cm deep) on lateral femoral condyle with osteochondral loose body

A 31-year-old female patient was referred to the senior author after failed conservative treatment of painful right knee for 7 months. She had no known history of knee injury. At age 30 years, about 7 months before presentation to the senior author, the patient began to experience intermittent right knee pain, popping, giving way and locking, which was not improved by conservative treatments including medications and physical therapy. On presentation, the patient had disabling knee pain with walking at the anterolateral aspect, which was aggravated with ascending or descending stairs. Physical examination revealed significant lateral joint line tenderness with positive McMurray test [28]. She also had snapping on the lateral compartment on knee motion. Plain radiographs (Fig. 1) and magnetic resonance imaging (MRI) (Fig. 2) revealed a large osteochondral defect of approximately 27 mm × 22 mm in size and 15 mm deep on the lateral femoral condyle with osteochondral loose bodies (x 3). Complex tear of lateral meniscus was also found. Therefore, in addition to arthroscopic loose body removal and lateral partial meniscectomy, the osteochondral lesion should also be treated. Considering the size and depth of the lesion, as well as her age, the patient was not a good candidate for microfracture, OAT or ACI as described above. A transplantable osteochondral allograft was not available. Thus, a novel therapeutic option, transplantation of a composite of UCB-MSCs and HA hydrogel, was planned in this case. The UCB-MSCs and HA hydrogel composite was produced by a manufacturing company (Medipost Inc., Seoul, South Korea) under regulatory authority approved good manufacturing practice (GMP) guidelines [22–24]. The UCB-MSCs were isolated and characterized according to previously published methods [29]. This study was approved by the institutional review board at our institution. Informed consent was obtained from patient included in the study.

**Fig. 3:**
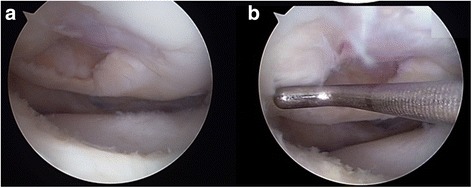
**a, b** Arthroscopic views from the anteromedial portal shows large osteochondral defect on lateral femoral condyle

**Fig. 4:**
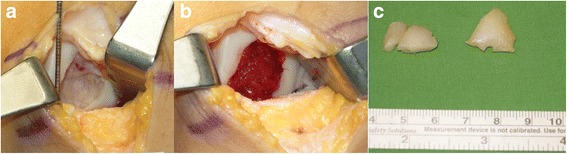
Gross photos shows **a** initial osteochondral defect site, **b** defect site just after implantation of umbilical cord blood derived mesenchymal stem cells, and **c** removed loose bodies

The patient was taken to the operating room, where spinal anesthesia was induced. An arthroscopic examination was performed using a standard anterolateral portal in supine position. After complete inspection of the joints and assessment of the defects (Fig. 3), a standard anteromedial portal was made and three osteochondral loose bodies were removed. Additionally, partial meniscectomy was performed for the complex tear of the lateral meniscus. An arthrotomy through an incision of approximately 3 cm in length was made through the anterolateral portal. The osteochondral defect on the lateral femoral condyle was carefully debrided down to the bed of the defect with a curette until healthy looking underlying bone appeared. Subsequently, multiple drilling with a 5 mm diameter drill bit was performed to the depth of 5 mm for the containment of the composite of UCB-MSCs and HA hydrogel. After the drilling, irrigation was performed to wash out the debris of bone and cartilage and the lesion site was dried using suction and gauze for implantation. Finally, the composite of UCB-MSCs 0.5 x 10^7^/ml and 4% HA hydrogel taken and filled in a 5 mL syringe. Then, the hydrogel mixture was implanted into the 5 mm drill holes from the base to the surface by slow injection to avoid any void (Fig. 4). As the hydrogel is not sticky, the 5 mm deep drill holes mainly served for the containment of the implanted MSC-hydrogel mixture. Actually, the small amount of blood smearing into the hydrogel seemed to form a clot intermingled with the hydrogel, thus help maintain the hydrogel in place. After the implantation, the knee was extended carefully with some retraction of capsular tissues to avoid displacement of the overlying composite of UCB-MSCs and HA hydrogel from the lesion. The wound was closed and a cylinder splint was applied. The patient started continuous passive motion exercises on postoperative day 1 and was ambulatory with crutches. Non-weight bearing ambulation was recommended until 3 months postoperative and gradually increasing weight bearing as tolerable was allowed thereafter.

**Fig. 5:**
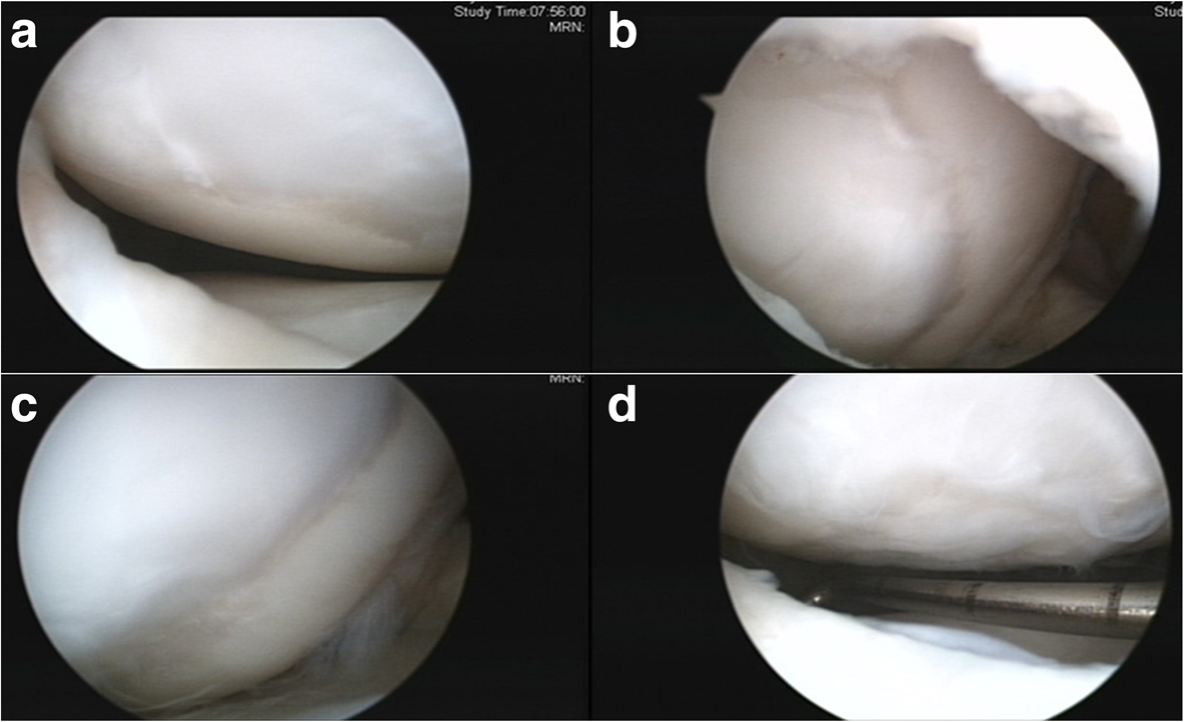
**a, b, c, d** Second look arthroscopy shows cartilage repair on lateral femoral condyle at postoperative 1 year

**Fig. 6:**
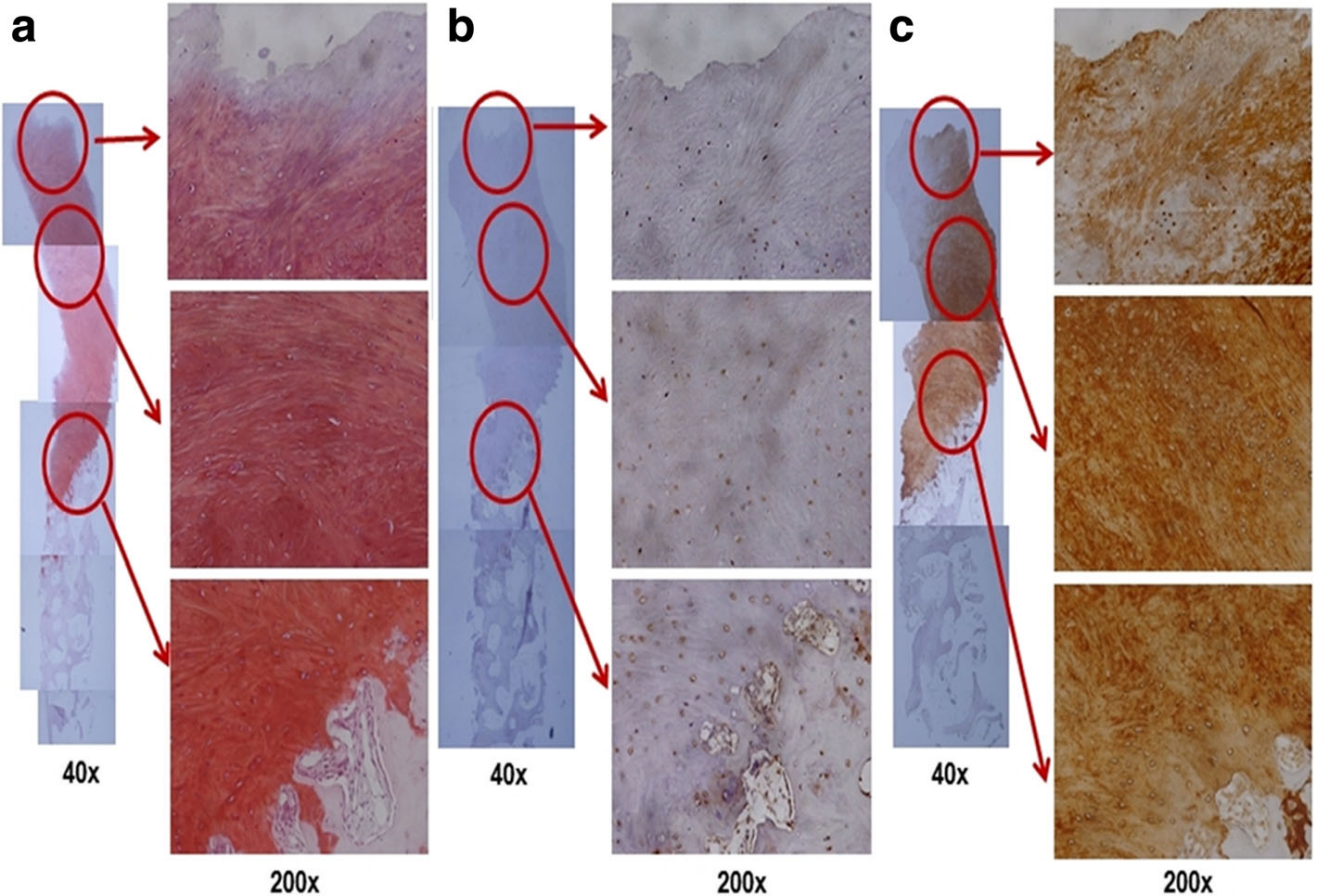
Histological findings. **a** Positive safranin – O staining was observed throughout the matrix. Immunostaining showed **b** weak staining for type I collagen but **c** diffuse strong positivity for type II collagen

**Fig. 7:**
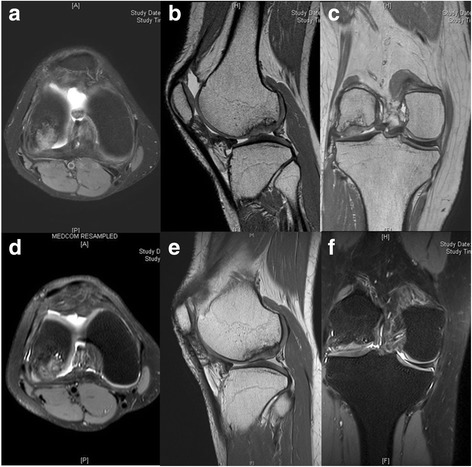
Magnetic resonance image. **a, b, c** The repair of the osteochondral defect at postoperative 1 year was observed and **d, e, f** the repaired tissue was maintained for 5.5 years without deterioration

Pain on walking by 100 mm visual analog scale (VAS) was improved from 46 preoperatively to 8 at postoperative 1 year. The international knee documentation and committee (IKDC) subjective score improved from 63.22 preoperatively to 85.02 at postoperative 1 year. The Western Ontario and McMaster Universities Osteoarthritis Index (WOMAC) score improved from 25 preoperatively to 2 at postoperative 1 year. Second look arthroscopy and biopsy from the implantation site were performed at postoperative 1 year after informed consent. The site of previous large chondral defect was smooth and fully covered with hyaline-cartilage like tissue, which was generally firm-to-hard with excellent peripheral integration (Fig. 5). There was no area of bone formation or bone exposure at the articular surface. Biopsy was taken with a biopsy needle, and histologic evaluation revealed evidence of hyaline-like cartilage regeneration. Positive Safranin-O staining was observed throughout the matrix suggesting the abundant presence of glycosaminoglycan, which is typical to hyaline cartilage matrix (Fig. 6a). With immunohistochemistry for type I collagen and type II collagen, typical for fibrocartilage and hyaline cartilage, respectively, weak positivity for type I collagen (Fig. 6b) and diffuse strong positivity for type II collagen was observed (Fig. 6c). MRI at postoperative 1 year showed good filling of the defect with abundant repair tissue and smooth integration to surrounding tissue (Fig. 7a-c). Moreover, the deep portion of the previous defect corresponding to underlying bone was partially restored as bony tissue, while the superficial portion near the articular cartilage was restored as cartilagenous tissue.

**Fig. 8:**
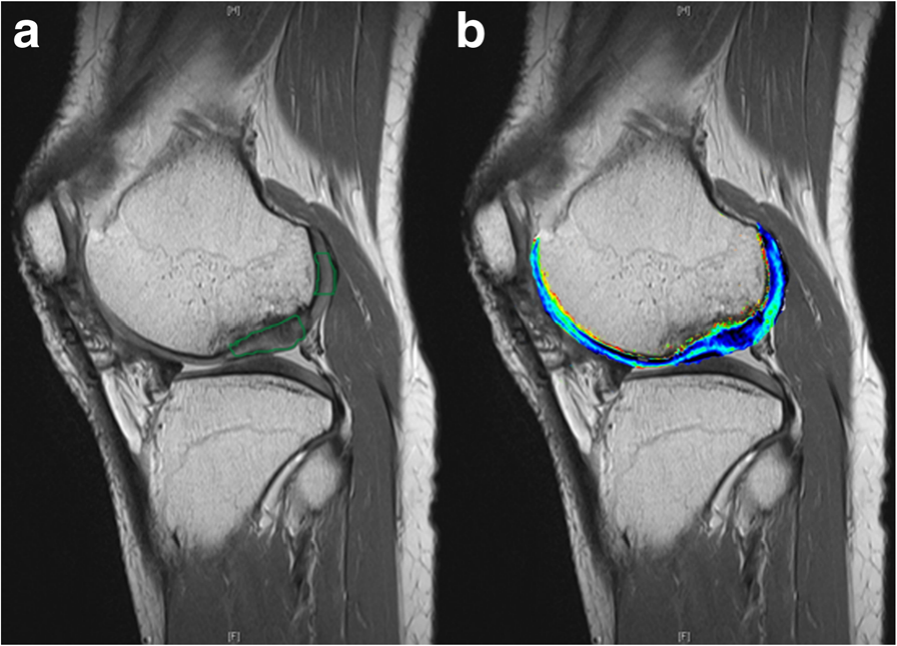
**a** The change in quantitative R1 in regenerated cartilage and in native cartilage were obtained at the marked areas to calculate the relative R1 index, which equals 1.0 in the case of perfect regeneration. **b** Higher T1 values (marked in blue) were associated with increased relative GAG content, which was observed in regenerated cartilage

The improved scores were maintained until the latest follow up at 5.5 years postoperatively with VAS 12, IKDC 85.05 and WOMAC 4. The MRI performed at 5.5 years after surgery showed maintenance of the repair tissue with filling of the defect and integration to surrounding tissue (Fig. 7d-f). A delayed gadolinium-enhanced MRI of the cartilage [30] indicated high glycosaminoglycan content of the regenerated cartilage (relative ΔR1 index = 1.41, Fig. 8). The restored bony tissue in the deep portion and the restored cartilage tissue in the superficial portion were maintained without deterioration or transition to bony tissue. During follow-up period, no specific adverse reactions were observed until 5.5 years.

## Discussion

We report a case of a successful outcome using the composite of UCB-MSCs 0.5 x 10^7^/ml and 4% HA hydrogel for the treatment of large and deep osteochondral defect of the knee. The clinical results at 1 year and at 5.5 years postoperatively suggest that this method can be a viable option in restoring large and deep osteochondral defects. To our knowledge, this is the first report of a successful treatment of large osteochondral defect of a human joint by application of allogeneic MSCs-based product.

Over the past decade, clinical and basic research has provided the foundation for successful treatment of focal cartilage defects [31, 32]. The main approaches currently used in clinical practice are microfracture, OAT, and ACI. Microfracture and OAT are generally known that they not recommended for large lesions [33, 34]. ACI was the first cell therapy used clinically to treat cartilage defects [35]. ACI has undergone several improvements over time [36, 37]. However, there are still several shortcomings even with the newly developed ACI techniques; the main shortcoming is an age-related chondrocyte de-differentiation during the expansion phase [38]. Chondrocyte is known to dedifferentiate to a fibroblast-like state during cultivation in monolayers. The dedifferentiation represents both morphological changes and alterations in collagen expression patterns, which negatively affects the potential of the implanted cells to restore the cartilage tissue. In addition, ACI requires autologous bone implant for the restoration of the subchondral bone in large osteochondral defects [11, 12].

To overcome the limitation and shortcomings of currently available options, a novel option seems to be required for the treatment of large osteochondral lesion of the knee. In this regard, the use of MSCs can be a potential therapeutic option for the restoration of cartilage as well as bone in the osteochondral defects, considering the MSCs’ capacity of self-renewal, multi-lineage differentiation potential and immunomodulation [39]. Several studies reported that MSCs with scaffold can repair osteochondral defects in animal models [40–43]. We have already experienced successful restoration of osteochondral defects with no immunologic problem after transplantation of human UCB-MSCs in an animal model which was a xenograft model [22–26]. Also, we had seen the safety and efficacy of UCB-MSCs for the restoration of articular cartilage defect in seven osteoarthritic patients in phase 1/2 clinical trial performed at our institution [27]. Therefore, we tried to extend the novel approach to the restoration of subchondral bone as well as the articular cartilage in large osteochondral defect case, and the result was encouraging without any significant adverse events.

To our knowledge, this is the first case report of the transplantation of allogeneic MSCs for the restoration of large osteochondral defects of the human knee. In the literature, there have been only two previous studies which used autologous MSCs or mixed cell concentrate containing MSC for the treatment of osteochondral defect of the human knee [16, 17]. A case report of one patient with a 1-year follow-up presented that the restoration of articular cartilage and subchondral bone for an osteochondral defect was promoted by implantation of autologous bone marrow (BM)-MSCs embedded in calcium hydroxyapatite ceramic with interconnected pores [16]. The technique required two-stage surgery and invasive BM collection. The other study with a 2-year follow-up described the use of BM aspirate concentrate (BMAC), a mixture of heterogeneous cell populations, embedded in hyaluronan based scaffold for osteochondral defects of the knee in 20 patients [17]. Clinical outcomes were improved and MRI showed bone and cartilage growth, nearly complete defect filling and satisfactory integration with surrounding tissue in 80% of patients at 1 year. Histological staining showed the presence of proteoglycan, particularly in the middle and deep zone. Unfortunately, the images of immunohistochemical staining for type 1 and type 2 collagen were not provided. Although this technique was one-stage surgery, it also required invasive BM aspiration for cell collection. In addition, heterogeneous cell populations had been used in this study. The MSCs are known to be present in less than 0.1% of BM aspirate concentrates [44]. Thus it is difficult to determine whether the bone and cartilage repair was by the MSCs or other components, such as platelet derived growth factors, and consistent results could not be expected. Moreover, these two related previous reports lacked longer term follow-up to evaluate whether the restored tissues were maintained and provided reliable and durable clinical outcomes. We believe the results of the case in the current report warrant further investigations on the application of allogeneic MSCs for the restoration of osteochondral defects.

In this case report, the improvements in pain and function at 1-year post-transplantation were maintained for 5.5 years. At the latest clinic visit of 5.5 years postoperatively, she had returned to full activity without any limitation as a nurse in a local hospital. In MRI and second-look arthroscopy at postoperative 1 year, no overgrowth, delamination or fibrous degeneration at the site of newly formed tissue were observed, which is often observed after ACI [45]. In addition, MRI at 5.5 years after surgery showed the maintenance of restored subchondral bone as well as the overlying articular cartilage with excellent peripheral integration. We think that the restoration of subchondral bone which provide a sound biomechanical environment for the restored defect site as well as the restoration of good quality cartilage should have contributed to the observed durable improvement in pain and function. The result of this case suggests that the transplantation of the composite of UCB-MSCs and HA will be an effective therapeutic option for the treatment of large osteochondral defects of the knee.

There was no adverse effect for 5.5 years. No abnormal findings suggesting rejection, foreign body reaction, or differentiation towards other mesenchymal lineage was observed. UCB-MSCs showed low immunogenicity and immunomodulatory activity [46, 47]. Other *in vivo* studies using UCB-MSCs have shown no immune rejection [22, 23, 25]. One recent study reported that UCB-MSCs transplanted cells disappeared at 4–8 weeks [48], which may contribute to the safety of transplantation of allogeneic UCB-MSCs in this case.

Some limitations of this study needs to be addressed. First, allogeneic MSCs transplantation might induce an immune reaction. However, the UCB-MSCs show low immunogenicity, and have immunomodulatory activity [47, 49]. In addition, previous *in vivo* studies using UCB-MSCs have not shown an immune rejection [22–24, 27]. In this study, there was no adverse reaction resulting from the rejection response. Second, the lateral meniscectomy and removal of intra-articular osteochondral loose bodies have also contributed for the improvement of the pain and function of the patient. However, we believe that the improvement could have not been that much as in this case without repair of the large and deep osteochondral defect. Third, meniscal loss (especially at the lateral compartment) has been considered as the contraindication of cell-based cartilage repair. However, we demonstrated that transplantation a composite of allogeneic UCB-MSCs and HA hydrogel was safe and effective modality for cartilage repair in osteoarthritic knees in which meniscal loss was combined, which was maintained more than 7 years without deterioration or significant adverse events [27]. Therefore, we believe that transplantation of the composite of UCB-MSCs and HA hydrogel is an appropriate modality for cartilage repair even though patients have an meniscal problem. Fourth, we could not rule out the effect of HA in the restoration of the osteochondral defect, although the HA hydrogel was used for delivering the MSCS and holding the MSCs in place. However, we learned from the preclinical studies using HA hydrogel with or without UCB-MSCs that the role of HA hydrogel in restoring the articular cartilage defect had been limited and the composite of UCB-MSCs and HA hydrogel showed consistently better results [22, 24]. Fifth, with the result of this case, we cannot tell whether the result of UCB-MSCs transplantation is better than ACI-collagen or matrix-associated ACI [50]. However, considering the fact that the integrity of the subchondral bone is important for a long term integrity of the overlying articular cartilage due to the biomechanical environment issue [51, 52], we believe that the novel option we report here will be more suitable than ACI or its modifications. Finally, this case may need an even longer term outcome.

## Conclusion

The results of this study showed that the transplantation of the composite of UCB-MSCs and HA hydrogel can be a viable therapeutic option for the restoration of large osteochondral defects of the human joint. It can be performed through a one-stage arthroscopy assisted surgery with a small arthrotomy. The result of this case report warrants further studies on this novel therapeutic option.

## Abbreviations

ACI: Autologous chondrocyte implantation
BM: Bone marrow
GAG: Glycosaminoglycan
MACI: Matrix-associated ACI
MRI: Magnetic resonance imaging
MSC: Mesenchymal stem cell
OAT: Osteochondral autograft transfer
UCB: Umbilical cord blood
